# Malignancies in the inborn errors of immunity

**DOI:** 10.1590/1806-9282.2024S104

**Published:** 2024-06-07

**Authors:** Ekaterini Simões Goudouris, Mara Morelo Rocha Felix, Fábio Chigres Kuschnir, Dirceu Solé

**Affiliations:** 1 Universidade Federal do Rio de Janeiro – Faculty of Medicine, Instituto de Puericultura e Pediatria Martagão Gesteira, Scientific Department of the Brazilian Association of Allergy and Immunology – São Paulo (SP), Brazil.; 2 Universidade Federal do Estado do Rio de Janeiro – School of Medicine and Surgery, Federal Hospital of State Servants, Research Department of Brazilian Association of Allergy and Immunology – São Paulo (SP), Brazil.; 3 Universidade Estadual do Rio de Janeiro – Faculty of Medical Sciences, President Brazilian Association of Allergy and Immunology – São Paulo (SP), Brazil.; 4 Universidade Federal de São Paulo – São Paulo School of Medicine, Research Department of Brazilian Association of Allergy and Immunology – São Paulo (SP), Brazil.

## INTRODUCTION

Inborn errors of immunity (IEI) are a group of approximately 500 diseases with genetically determined changes in the immune system's development and/or function. As a group, they are considered rare diseases, affecting 1 in 2,000 individuals on average^1^.

According to the periodically updated International Union of Immunology Societies (IUIS) classification, these diseases are distributed into 10 groups: combined T and B cell deficiencies; combined T and B cell deficiencies associated with syndromes; predominantly antibody deficiencies; diseases of immune dysregulation; congenital defects of phagocyte number or function; deficiencies in intrinsic and innate immunity; autoinflammatory diseases; complement system deficiencies; diseases with bone marrow failure; and phenocopies of immunodeficiencies. The most common defects worldwide are deficiencies in antibody production, with selective immunoglobulin A (IgA) deficiency being the most common oligo or asymptomatic defect and common variable immunodeficiency, the most frequent symptomatic defect^2^.

The main clinical manifestations of this group of diseases, particularly the most classic defects, formerly called primary immunodeficiencies, are infections that can be repeated and/or severe, requiring venous antibiotics to resolve them, caused by common or opportunistic microorganisms. The type of infectious agent and the location of infections are related to the sector of the immune system most affected by each disease. Some infections are very characteristic of some diseases and are called sentinel infections^3^.

In recent years, with the advent of genetic sequencing, the number of IEI described has increased dramatically, many of which are associated with manifestations of dysregulation of the immune system: allergies, autoimmunity, autoinflammation, benign lymphoproliferation, and malignancies^4^. Many patients begin the clinical picture of their diseases with these noninfectious manifestations, so if we use only infections as warning signs for the suspicion of an IEI, we may lose 25% of early diagnoses^5^.

The loss of immune surveillance capacity, with the recognition and elimination of emerging tumor cells, is the mechanism most easily remembered to justify the risk of malignancies in IEI. However, there are other recognized mechanisms^6^.

Our objective in this non-systematic literature review is to present the main mechanisms related to the development of malignancies in IEI and describe the most common malignancies found in the IEI group and associated with different types of IEI.

## MECHANISMS RELATED TO PREDISPOSITION TO MALIGNANCIES IN INBORN ERRORS OF IMMUNITY

We can list four intrinsic mechanisms related to the risk of developing malignancies in several IEIs, usually involving the cell type affected by the disease. These mechanisms are not exclusive and can act simultaneously in diseases^6,7^.

1. defects in the development of stem and myeloid cells;
2. defects in lymphocyte development, differentiation, and apoptosis;
3. deficiencies in the co-signaling, cytoskeleton, cytotoxicity, or metabolism of lymphocytes; and
4. defects in DNA repair, telomere maintenance, and chromosomal stability.

Three extrinsic mechanisms of oncogenesis are relevant in some IEIs, usually involving cell types not primarily affected by the immune defect^6,7^:

1. viral infections;
2. chronic tissue inflammation; and
3. impaired immune surveillance.

In Table 1, we list some examples of malignancies and IEI for each of the oncogenic mechanisms described.

**Table 1:** Examples of malignant diseases and inborn errors of immunity associated with oncogenesis mechanisms.

Type of malignancies	Inborn errors of immunity	Main mechanisms of oncogenesis
MDS, AML	Congenital neutropenias Chédiak-Higashi syndrome	Defects in the development of stem and myeloid cells
Lymphomas, leukemias, HLH	CVID, ALPS	Defects in lymphocyte development, differentiation, and apoptosis
	CVID, combined deficiencies of T and B cells	Defects in co-signaling, cytoskeleton, cytotoxicity, or lymphocyte metabolism
Lymphomas, leukemias, carcinomas, sarcomas	Ataxia telangiectasia, nijmegen syndrome, combined deficiencies of T and B cells, congenital dyskeratosis	Defects in DNA repair, telomere maintenance, and chromosomal stability
Lymphomas, leukemias, carcinomas, sarcomas, HLH, smooth muscle tumor	WHIM, epidermodysplasia verruciformis, combined deficiencies of T and B cells	Viral infections
Carcinomas	Innate immunity defects, IBD, virtually any IEI	Chronic tissue inflammation
	Adaptive immunity defects, virtually any IEI	Impaired immune surveillance

MDS: myelodysplastic syndrome; AML: acute myeloid leukemia; CVID: common variable immunodeficiency; ALPS: autoimmune lymphoproliferative syndrome; HLH: hemophagocytic lymphohistiocytosis; WHIM: warts, hypogammaglobulinemia, infections, and myelocathexis; IBD: inflammatory bowel disease; IEI: inborn errors of immunity. Adapted from Hauck et al.^6^.

## MAIN MALIGNANT DISEASES IDENTIFIED IN INBORN ERRORS OF IMMUNITY

The malignancies most identified in IEI are those related to the lymphoreticular system: lymphomas, leukemias, malignant histiocytosis, and thymus tumors. According to a survey conducted with data from the North American registry (USIDNET), these hematological malignancies corresponded to approximately 96% of the identified malignancies. Other malignant diseases corresponding to 36% of the tumors identified were skin, genitourinary, gastrointestinal, and breast cancer^8^.

The overall incidence of cancer is increased by 1.42 times, being 1.91 times in males and 1.12 times in females. In patients with IEI, the pediatric age group and adults between 40 and 50 years of age are affected more often than expected^8^.

Malignant diseases were the first clinical presentation in 0.8% of cases of IEI, especially between 40 and 50 years old and in ataxia telangiectasia and activated phosphoinositide 3-kinase-delta syndrome (APDS)^8^.

The risk of skin cancer is 4.55 times higher than expected in men and 3.33 times higher in women. The risk of lymphoma in patients with IEI is 10 times higher in men than expected in different age groups, and in women, 8.34 times^8^.

The genetic signature identified in lymphomas in patients with IEI differs from those without IEI, and germline and somatic mutations were described. Somatic mutations in *BRWD3* identified in the lymphomas of a group of patients with APDS are relevant^9^.

A multicenter study in Turkey identified a prevalence of malignancies of 0.9% in patients with IEI, with a male:female ratio of 1.8, a median age at diagnosis of 10 years, and a mortality rate of 52.5%. Most patients were diagnosed with ataxia telangiectasia (32.2%), and non-Hodgkin lymphoma was the most common malignancy. The risk of malignancy, however, was higher in patients with DOCK8 deficiency^10^.

Lymphoid malignancies in patients with IEI are more challenging to diagnose, especially because of previous persistent lymphoproliferation. There is also less response to treatment protocols, as well as an increased risk of toxicity related to them, which increases the complexity of the therapeutic approach for these patients. Radiotherapy is contraindicated in IEI when there is a defect in DNA repair with radiosensitivity, such as ataxia telangiectasia, Nijmegen syndrome, or combined T and B defects caused by a LIG4 mutation. Previous organic damage by the underlying disease, such as bronchiectasis, may also compromise the response to treatment^9^.

A systematic review of lymphomas in IEI patients showed that T cell defects were the most associated with lymphomas (57%), with a median age of diagnosis between 9.5 and 12 years. The most common type was diffuse large B-cell lymphoma (33.5%). Lymphomas related to the Epstein-Barr virus were found more frequently in innate immunity deficiencies. The complete response to treatment occurred in 65.8%, with death reported in 38.2% of cases^11^.

## INBORN ERRORS OF IMMUNITY MOST OFTEN ASSOCIATED WITH MALIGNANCIES

In several studies, the number of described malignancies is higher in predominantly antibody production deficiencies, especially in the common variable immunodeficiency, the most common symptomatic IEI worldwide. The prevalence of lymphoma, gastric, and breast cancer in individuals with CVID was 4.1, 1.5, and 1.3%, respectively, in a study with 8,123 patients^12^.

Other IEI commonly described in studies on malignancies and primary immune diseases are the combined deficiencies of T and B cells (ataxia telangiectasia mainly), APDS, hyper-IgE syndromes, Wiskott-Aldrich syndrome, and autoimmune lymphoproliferative syndrome (ALPS)^13–15^.

## MAIN MALIGNANT DISEASES ASSOCIATED WITH DIFFERENT TYPES OF INBORN ERRORS OF IMMUNITY

Table 2 shows the main types of cancer in several IEIs, and Table 3 shows the main IEIs to be considered for each kind of malignancy.

**Table 2:** Main types of cancer reported in some inborn immunity errors.

Inborn errors of immunity	Reported malignancies
Selective IgA deficiency	Gastric
	Lymphomas
CVID	Lymphomas (more frequently non-Hodgkin)
	Gastric
	Thymus
	Breast
	Bladder
	Cervical
X-linked agammaglobulinemia	Gastric
	Colorectal
Wiskott-Aldrich syndrome	Lymphoma
	Lymphoblastic leukemia
	Myelodysplasia-myeloproliferative disorders
22q11.2 deletion syndrome	Lymphoma
	Acute leukemia
Ataxia telangiectasia	Lymphoma
	Lymphoblastic leukemia
	Breast
	Liver
	Gastric
	Esophagus
	Glioma
WHIM syndrome	Lymphoma
	Genital and squamous carcinoma
	Acute myeloid leukemia

IgA: immunoglobulin A; CVID: common variable immunodeficiency; WHIM: warts, hypogammaglobulinemia, infections and myelocathexis. Adapted from Tiri et al.^13^.

**Table 3:** Main inborn errors of immunity to be considered according to the malignancy found.

Malignancies	Inborn errors of immunity
Non-Hodgkin and Hodgkin lymphomas, ALL	Combined deficiencies of T and B cells not severe
	Defects of DNA repair
	Predominantly antibody deficiencies
	Diseases of immune dysregulation
MDS, AML	Congenital neutropenia,
	Shwachman-Diamond syndrome,
	GATA2 deficiency,
	Diseases with bone marrow failure
CNS tumors	Defects of DNA repair
Solid tumors	Defects of DNA repair
	Congenital dyskeratosis (telomeropathies)
	PTEN deficiency (APDS-like)
	CVID
Smooth muscle tumors associated with EBV	Combined deficiencies T and B not severe
	Ataxia telangiectasia
	Deficiency of GATA2
	Deficiency of CARMIL2
	Deficiency of ZAP70
Kaposi's sarcoma	Wiskott-Aldrich syndrome
	XMEN syndrome
	Deficiency of IFNg receptor 1
	Deficiency of STIM
	Deficiency of OX40
Skin cancer, not melanoma	Epidermodisplasia verruciformis
	Deficiency of DOCK8
	Cartilage hair hypoplasia
	Xeroderma pigmentosum
	Chronic mucocutaneous candidiasis

ALL: acute lymphoblastic leukemia; MDS: myelodysplastic syndrome; AML: acute myeloid leukemia; CNS: central nervous system; EBV: Epstein-Barr virus; IFNg: interferon gamma. Adapted from Bosh et al.^16^.

## CONCLUSION

In general, malignancies have a higher incidence and are diagnosed at an earlier age in individuals with some IEI. There are several mechanisms of oncogenesis, transcending the simple impairment of immune surveillance and varying according to the type of defect in the immune system. Hematological malignancies are the most common, especially lymphomas, in patients with common variable immunodeficiency and defects in DNA repair. The response to treatment is worse than in individuals without IEI, with a higher risk of treatment-related toxicity and lower survival.
